# Supplementary material for: Abundance and diversity of resistomes differ between healthy human oral cavities and gut
Source: Nat Commun. 2020 Feb 4;11:693. doi: 10.1038/s41467-020-14422-w (PMC7000725; doi:10.1038/s41467-020-14422-w)
Supplement: Supplementary file 1 — Supplementary Information [file 41467_2020_14422_MOESM1_ESM.pdf]

Abundance and diversity of resistomes differ between healthy human oral cavities and gut

Carr et al.

Supplementary Material

Supplementary Figures

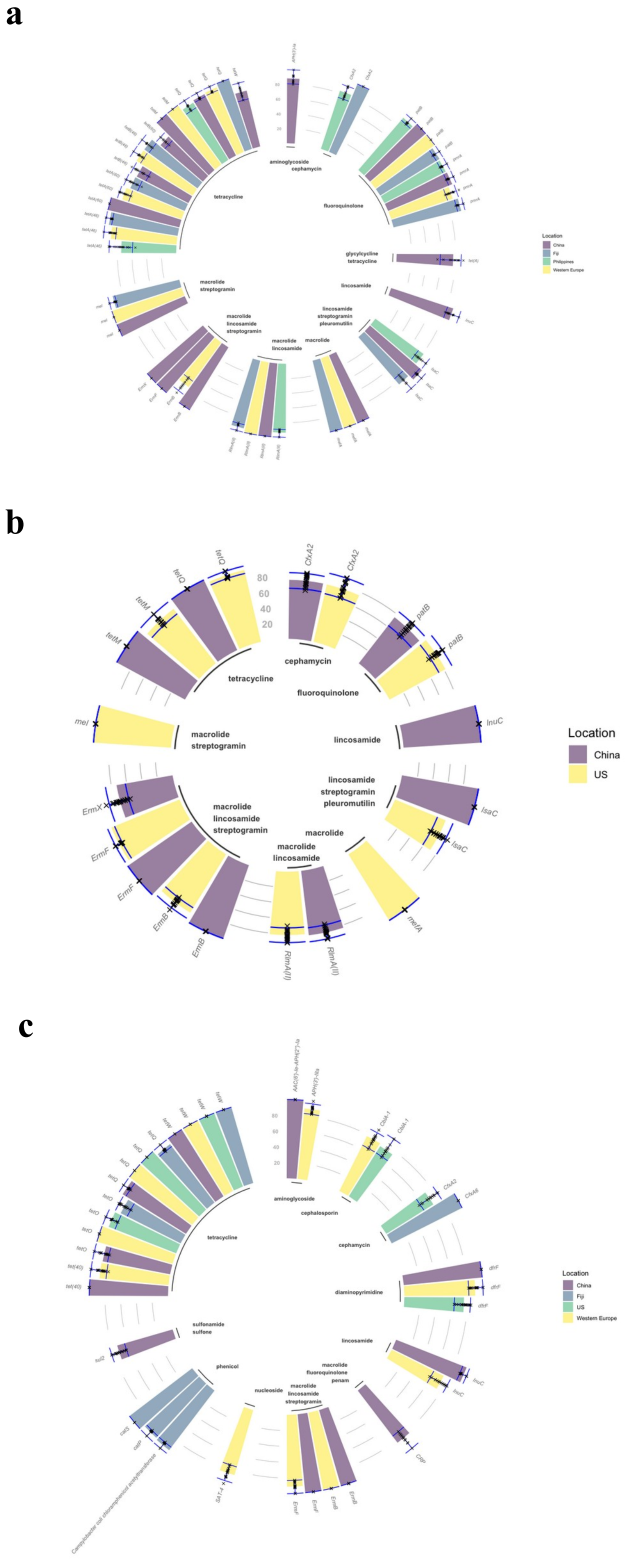

Supplementary Fig. 1 ARGs that are found >= 70% of individuals. a saliva from China (n = 18), Fiji (n = 18), the Philippines (n = 18) and Western Europe (n = 18), **b** dental plaque from China (n = 18) and the US (n = 18), and **c** stool from China (n = 18), Fiji (n = 18), the US (n = 18) and Western Europe (n = 18). The height of bars are the means and the error bars are 95 % confidence intervals (CIs) of percentages extracted from bootstrapping samples 20 times shown by points. Source data are provided in the Source Data file.

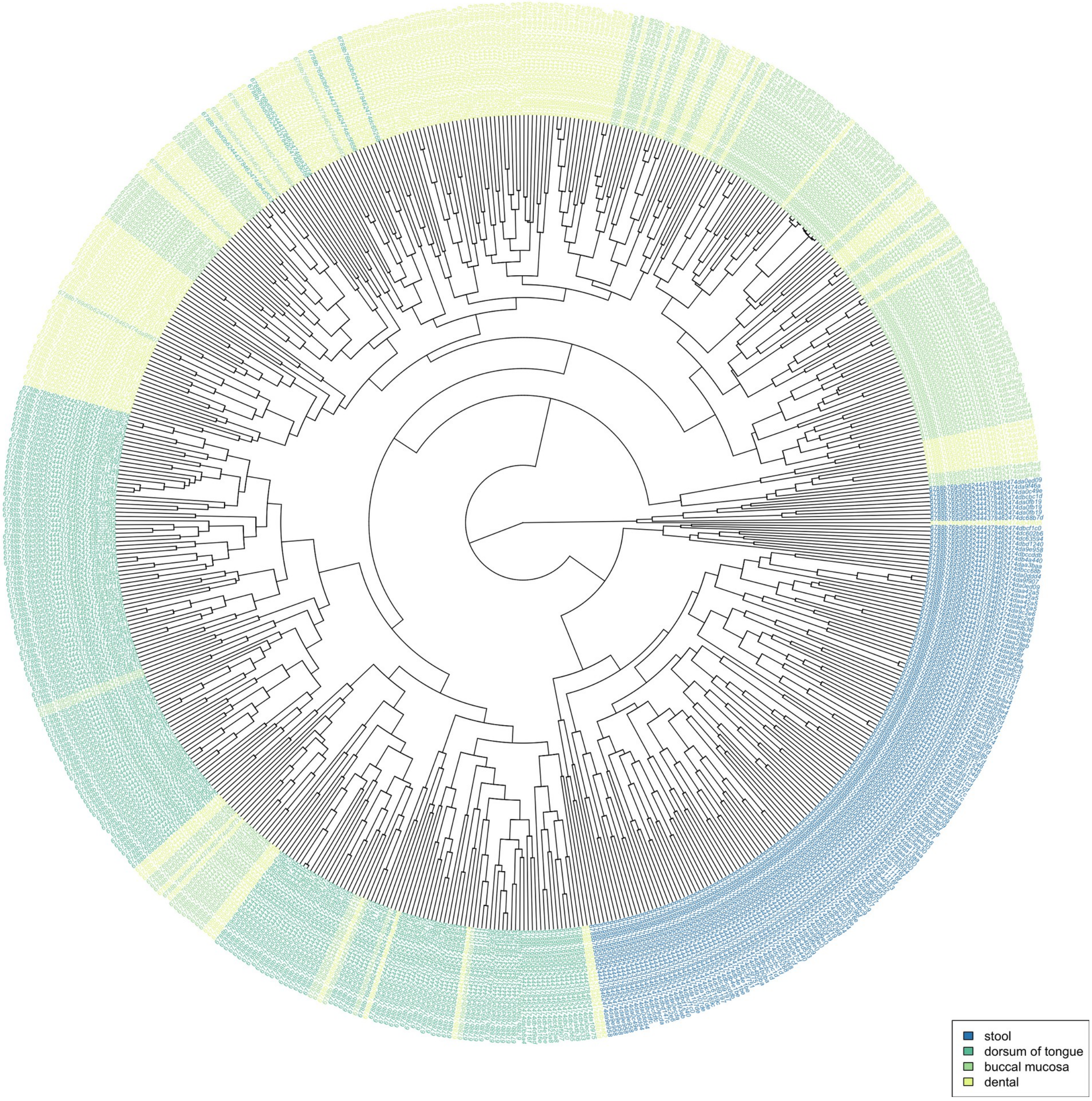

Supplementary Fig. 2 Longitudinal US samples clustered by ARG abundance profiles. Hierarchical clustering of ARG abundance (log10[RPKM+1]) (complete method on Euclidean distance matrix) and labelled by body site: buccal mucosa: n = 55 (36 with two, 18 with three and 1 with six timepoints), dorsum of tongue: n = 69 (43 with two, 24 with three and 2 with four timepoints), dental plaque: n = 67 (43 with two, 20 with three, 1 with four and 3 with six timepoints), stool n = 57 (33 with two, 21 with three, 2 with four and 1 with six timepoints). These samples were collected within two years with individuals having had no antimicrobial treatment in that time. Source data are provided in the Source Data file.

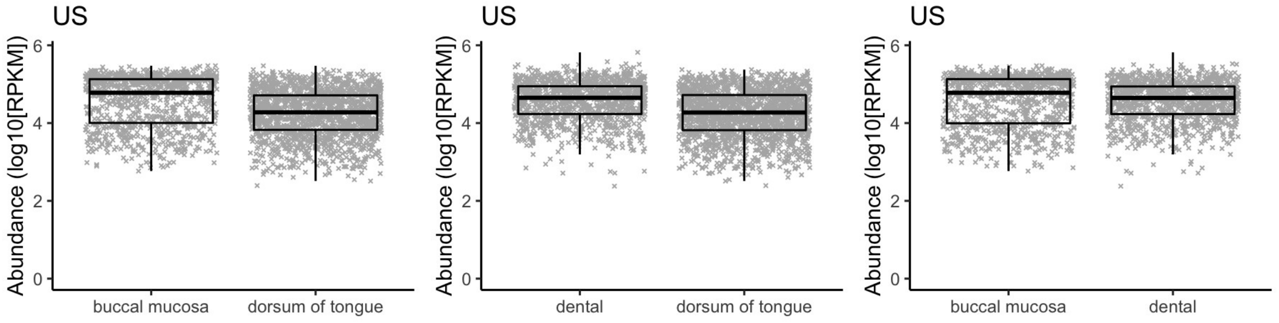

Supplementary Fig. 3 Comparing ARG abundance between oral cavity sites. Absolute abundance in log10 of reads per kilobase of read per million (RPKM) of ARGs for paired samples of individuals from the US (buccal mucosa and dorsum of tongue: n = 86, dental plaque and dorsum of tongue: n = 89, buccal mucosa and dental plaque: n = 86). Centre line is median, box limits are upper and lower quartiles, whiskers are 1.5x interquartile ranges and points beyond whiskers are outliers. Source data are provided in the Source Data file.

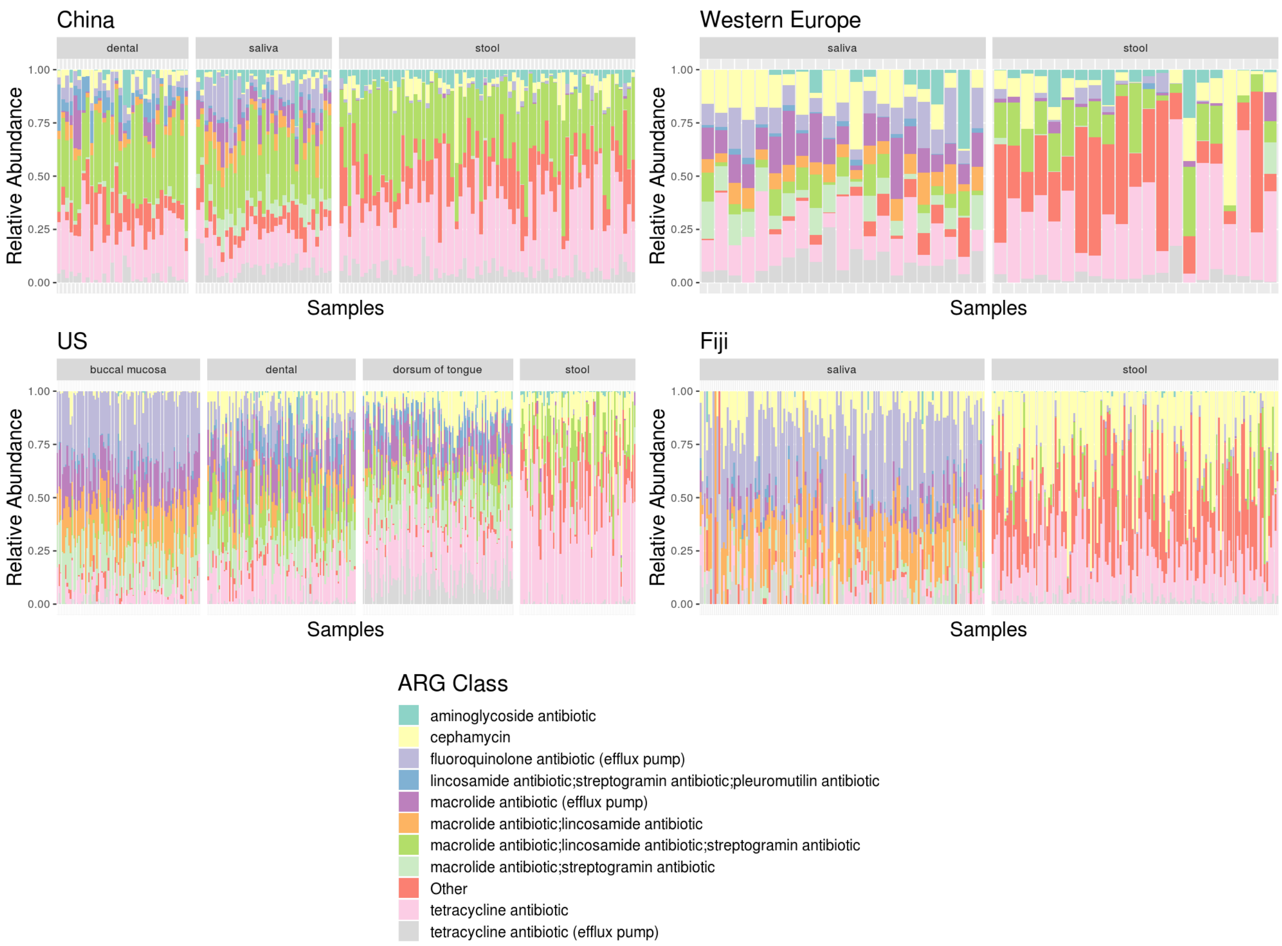

Supplementary Fig. 4 Comparing ARG abundance of different body sites between individuals. Relative abundance of reads labelled by the top ten most abundant ARG classes across all geographical locations or Other classes for each sample of individuals from China (saliva: n = 33, dental plaque: n = 32, stool: n = 72), Fiji (saliva: n = 136, stool: n = 137), the US (buccal mucosa: n = 87, dental plaque: n = 90, dorsum of tongue: n = 91, stool: n = 70) and Western Europe (saliva: n = 21, stool: n = 21). Source data are provided in the Source Data file.

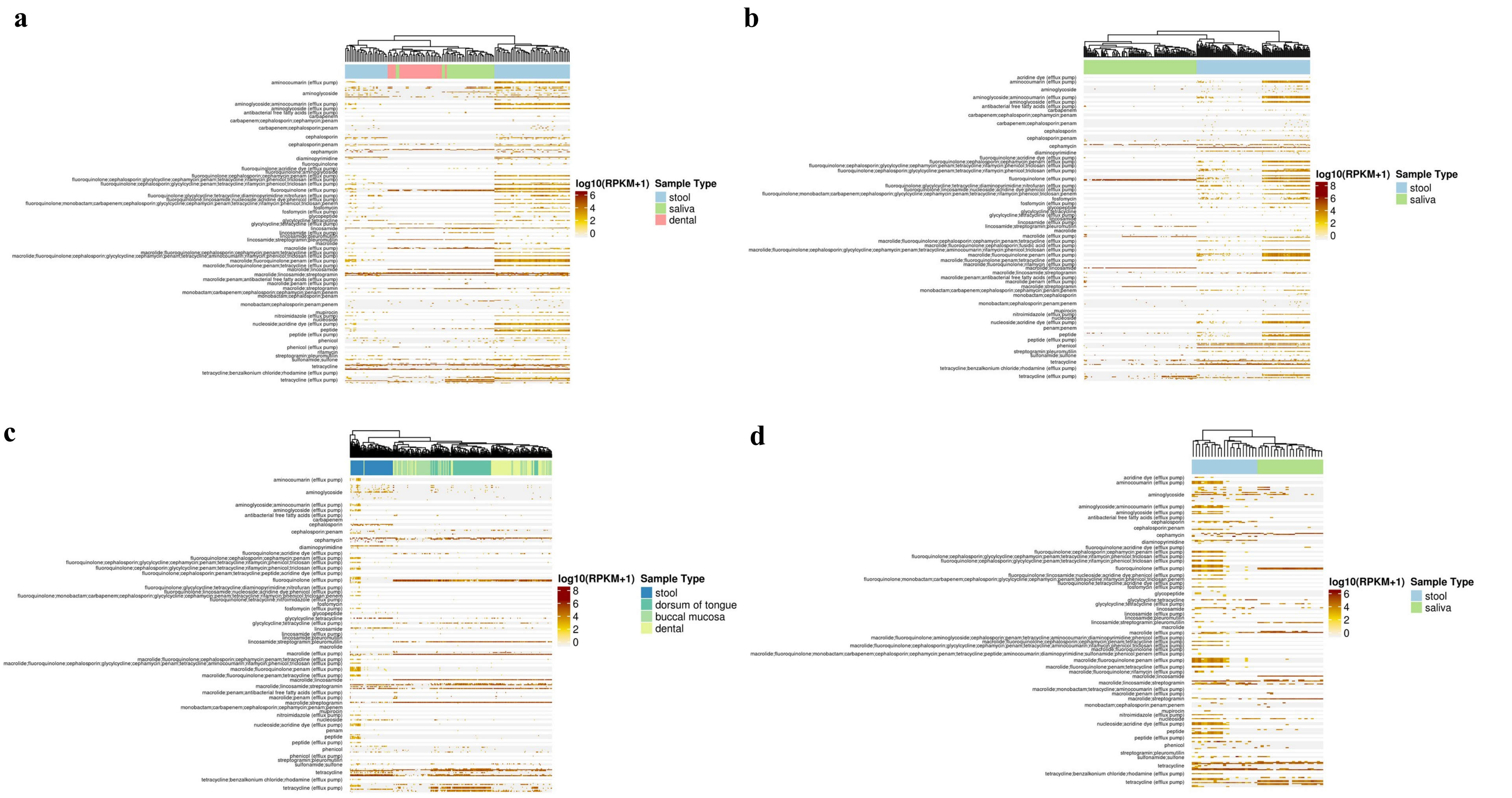

Supplementary Fig. 5 ARG abundance shown for each ARG and individual. Heatmaps of abundance (log10[RPKM+1]) clustered by hierarchical clustering column-wise by sample (complete method on euclidean distance matrix) and separated row-wise by ARG class for individuals from **a** China (saliva: n = 33, dental plaque: n = 32, stool: n = 72); **b** US including longitudinal samples (buccal mucosa: n = 164, dorsum of tongue: n = 188, dental plaque: n = 191, stool n = 156); **c** Fiji (saliva: n = 136, stool: n = 137); and **d** Western Europe (saliva: n = 21, stool: n = 21). Source data are provided in the Source Data file.

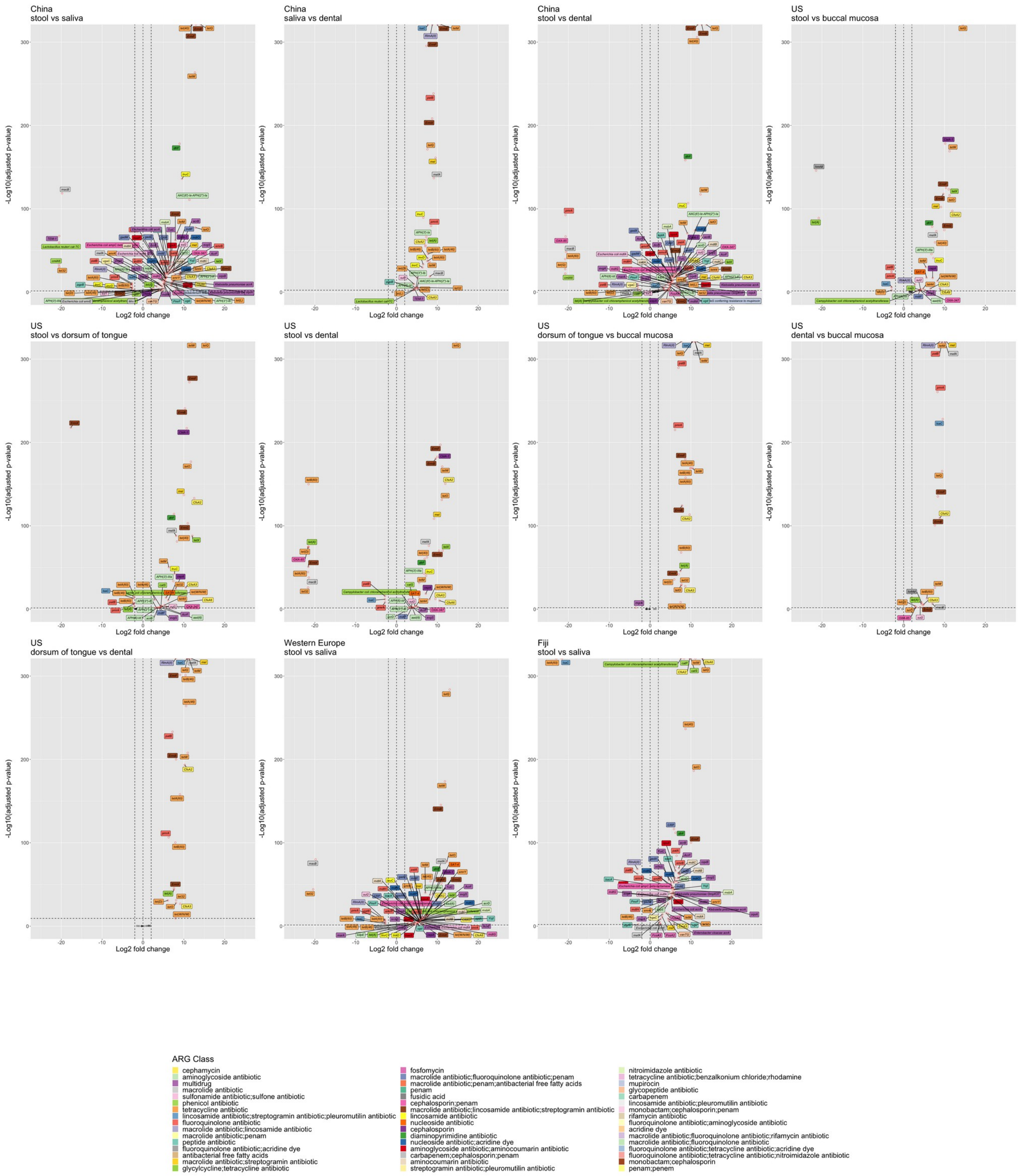

Supplementary Fig. 6 Differential analysis of ARG abundance between body sites for each geographical location. Volcano plots showing differential analysis, using DESeq2 package in R, between paired samples of adjusted p-value < 0.05 for individuals from China (stool and saliva: n = 31, dental plaque and saliva: n = 31, stool and dental plaque: n = 30), Fiji (saliva and stool: n = 132), the US (stool and buccal mucosa: n = 64, stool and dorsum of tongue: n = 69, stool and dental plaque: n = 68, dorsum of tongue and buccal mucosa: n = 86, buccal mucosa and dental plaque: n = 86, dorsum of tongue and dental plaque: n = 89) and Western Europe (saliva and stool: n = 21). Source data are provided in the Source Data file.

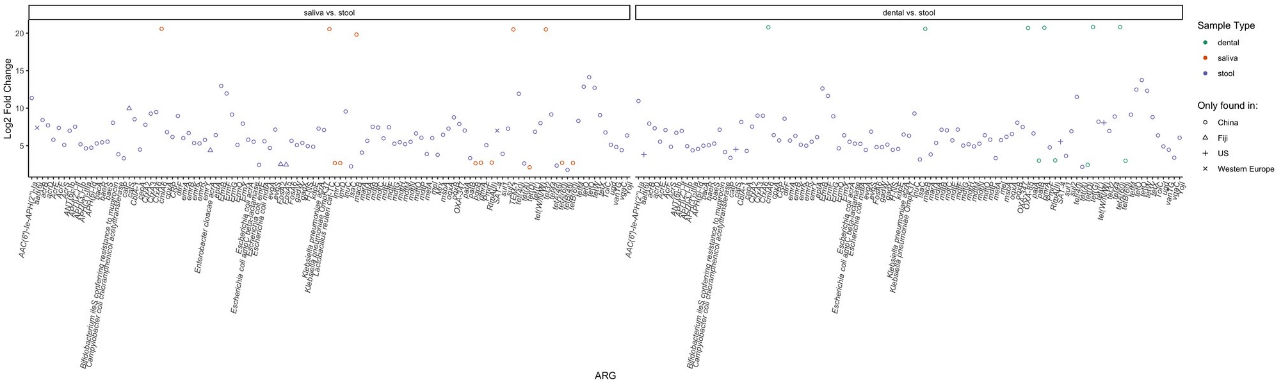

Supplementary Fig. 7 Log2 fold change of ARGs exclusively found in one geographical location between paired samples. ARGs selected where adjusted p-value < 0.05 from differential abundance analysis between paired samples of individuals from China (stool and saliva: n = 31, stool and dental plaque: n = 30), the US (stool and dental plaque: n = 68), Fiji (saliva and stool: n = 137) and Western Europe (saliva and stool: n = 21). Source data are provided in the Source Data file.

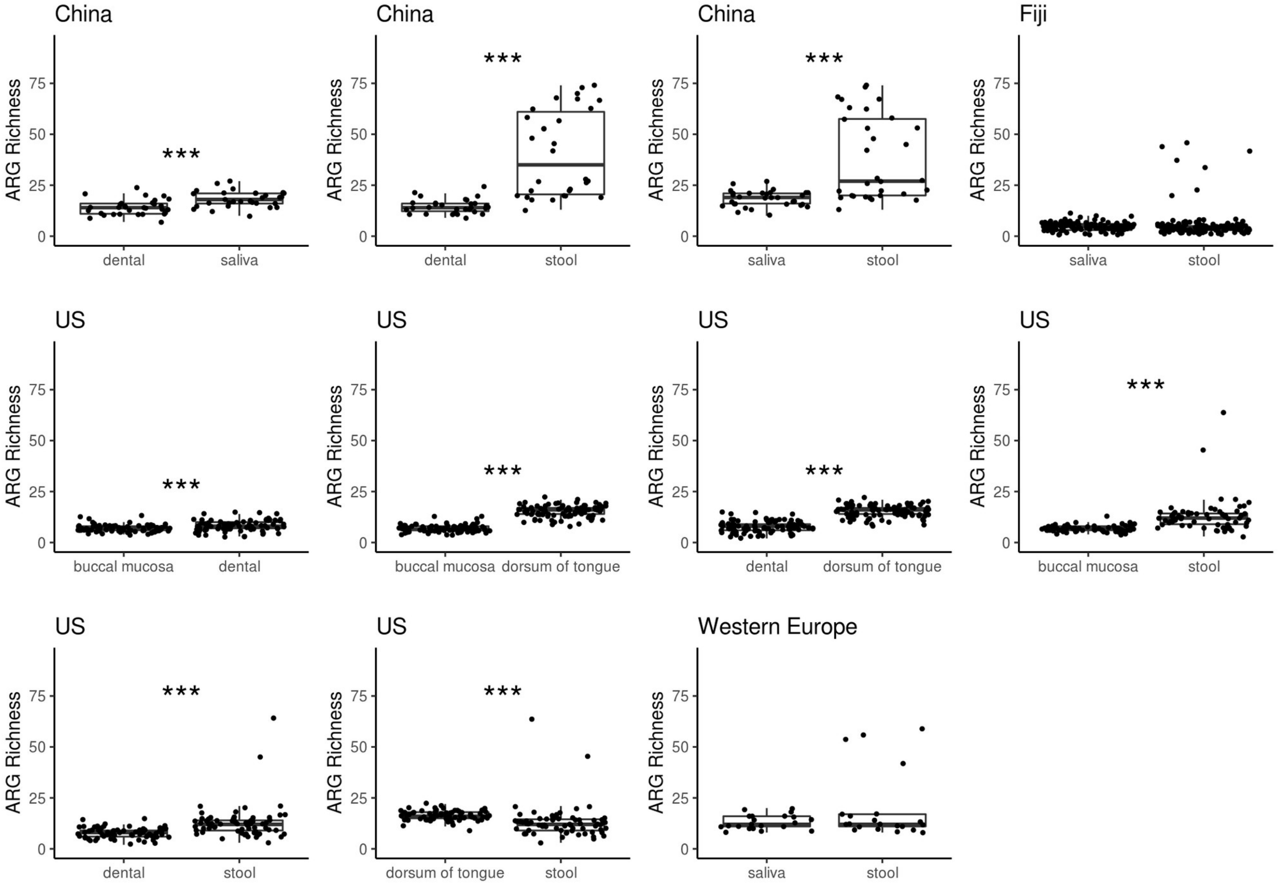

Supplementary Fig. 8 Comparing ARG richness between paired body sites excluding ARGs that are part of or regulate an efflux pump complex. ARG richness is defined as the number of unique ARGs that are not part of nor regulate an efflux pump complex for paired samples of individuals from China (dental plaque and saliva: n = 31, stool and dental plaque: n = 30, stool and saliva: n = 31), Fiji (saliva and stool: n = 128), the US (buccal mucosa and dental plaque: n = 78, buccal mucosa and dorsum of tongue: n = 86, dental plaque and dorsum of tongue: n = 89, buccal mucosa and stool: n = 64, dental plaque and stool: n = 68, dorsum of tongue and stool: n = 67) and Western Europe (saliva and stool: n = 21) with Mann-Whitney, paired, two-sided t-test (p-value < 0.05 as *, < 0.01 as **, < 0.005 as ***). Centre line is median, box limits are upper and lower quartiles, whiskers are 1.5x interquartile ranges and points beyond whiskers are outliers. Source data are provided in the Source Data file.

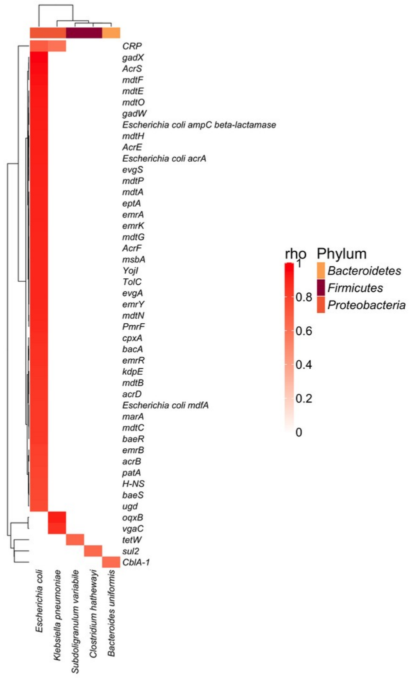

Supplementary Fig. 9 Spearman's correlation of ARG and species abundance from China stool samples. Samples of individuals (n = 31). Rows and columns are clustered by hierarchical clustering of Euclidean distance. Columns are coloured by phylum. P-values are adjusted by Benjamini-Hochberg multiple test correction. *Rho* shown only where adjusted p-value < 0.05. Source data are provided in the Source Data file.
