# Supplementary material for: Abundance and diversity of resistomes differ between healthy human oral cavities and gut
Source: Nat Commun. 2020 Feb 4;11:693. doi: 10.1038/s41467-020-14422-w (PMC7000725; doi:10.1038/s41467-020-14422-w)
Supplement: Supplementary file 3 — Description of Additional Supplementary Files [file 41467_2020_14422_MOESM3_ESM.docx]

**Description of Additional Supplementary Files**

File name: Supplementary Data 1

Description: Linear regression of ARG class abundance and antibiotic prescriptions

File name: Supplementary Data 2

Description: Cohort metadata
