# Supplementary material for: Abundance and diversity of resistomes differ between healthy human oral cavities and gut
Source: Nat Commun. 2020 Feb 4;11:693. doi: 10.1038/s41467-020-14422-w (PMC7000725; doi:10.1038/s41467-020-14422-w)
Supplement: Supplementary file 6 — Reporting Summary [file 41467_2020_14422_MOESM6_ESM.pdf]

nature research

Corresponding author(s): DBPR NCOMMS-19-10818A

Last updated by author(s): Dec 3, 2019

Reporting Summary

Nature Research wishes to improve the reproducibility of the work that we publish. This form provides structure for consistency and transparency in reporting. For further information on Nature Research policies, seeAuthors & Referees and theEditorial Policy Checklist .

Statistics

For all statistical analyses, confirm that the following items are present in the figure legend, table legend, main text, or Methods section.

n/a	Confirmed	
☐	☒	The exact sample size (*n*) for each experimental group/condition, given as a discrete number and unit of measurement
☐	☒	A statement on whether measurements were taken from distinct samples or whether the same sample was measured repeatedly
☐	☒	The statistical test(s) used AND whether they are one- or two-sided<br>*Only common tests should be described solely by name; describe more complex techniques in the Methods section.*
☐	☒	A description of all covariates tested
☐	☒	A description of any assumptions or corrections, such as tests of normality and adjustment for multiple comparisons
☐	☒	A full description of the statistical parameters including central tendency (e.g. means) or other basic estimates (e.g. regression coefficient) AND variation (e.g. standard deviation) or associated estimates of uncertainty (e.g. confidence intervals)
☐	☒	For null hypothesis testing, the test statistic (e.g. *F*, *t*, *r*) with confidence intervals, effect sizes, degrees of freedom and *P* value noted<br>*Give P values as exact values whenever suitable.*
☒	☐	For Bayesian analysis, information on the choice of priors and Markov chain Monte Carlo settings
☐	☒	For hierarchical and complex designs, identification of the appropriate level for tests and full reporting of outcomes
☐	☒	Estimates of effect sizes (e.g. Cohen's *d*, Pearson's *r*), indicating how they were calculated

Our web collection on statistics for biologists contains articles on many of the points above.

Software and code

Policy information about availability of computer code

Data collection	No software used
Data analysis	Open source software: FastQC 0.11.3, AlienTrimmer 0.4.0, Bowtie2 2.2.3, Bowtie2 2.2.5, CARD database 3.0.0, Samtools 1.9, Bedtools 2.28.0, seqtk 1.2, MetaPhlAn2 2.6.0, R 3.5.1, DESeq2 1.20.0 R package, metafor 2.1-0 R package, cluster 2.0.7.1 R package<br><br>Custom code: run_analysis.R in https://github.com/blue-moon22/resistomeData and resistomeAnalysis R package in https://github.com/

For manuscripts utilizing custom algorithms or software that are central to the research but not yet described in published literature, software must be made available to editors/reviewers. We strongly encourage code deposition in a community repository (e.g. GitHub). See the Nature Research guidelines for submitting code & software for further information.

Data

Policy information about availability of data

All manuscripts must include a data availability statement. This statement should provide the following information, where applicable:
- Accession codes, unique identifiers, or web links for publicly available datasets
- A list of figures that have associated raw data
- A description of any restrictions on data availability

ARG data, figures and tables are available at https://github.com/blue-moon22/resistomeData
High-resolution figures are available at https://figshare.com/s/5d020d6f52a7685f341f
Data underlying Figs 1-5 and Supp Figs 1-9 are provided in the Source Data file. All other data are available from the corresponding author upon reasonable requests.

Field-specific reporting

Please select the one below that is the best fit for your research. If you are not sure, read the appropriate sections before making your selection.

☒ Life sciences ☐ Behavioural & social sciences ☐ Ecological, evolutionary & environmental sciences

For a reference copy of the document with all sections, see nature.com/documents/nr-reporting-summary-flat.pdf

Life sciences study design

All studies must disclose on these points even when the disclosure is negative.

Sample size

No sample size calculation was performed in this study. The study relied on available open source datasets of five cohorts (China, Fiji, the Philippines, US and Western Europe) from seven previously published studies. The sample sizes for each cohort varied widely from 21 saliva/stool samples in Western Europe to 191 dental plaque in US. Generally all samples (including exclusion criteria) were used in analyses apart from:

Fig 1 and Sup Fig 1:
For the comparison of the proportion of samples containing ARG classes, mechanisms and ARGs between cohorts, we selected 18 samples randomly from each cohort and body site (saliva, dental and stool) to ensure equal sample sizes across cohorts and body sites.

(See Data Exclusions and Randomization for more details).

Data exclusions

Fig 1 and Sup Fig 1:
18 were chosen as a sample size because only 18 out of the 23 Philippines saliva samples had a read count of over 6.9 million, as described in Methods.

Fig 2a, b:
For clustering ARG incidence profiles into distinct groups, samples excluding longitudinal US samples that were above a suitable subsample threshold were included. 1 US buccal mucosa, 3 China dental, 10 US dental, 7 Fiji saliva, 1 Philippines saliva, 1 Fiji stool were excluded as they had lower than subsample threshold of 1 million reads.

Sup Fig 2:
Only longitudinal samples included samples of body sites that were taken two or more times at different visits.

Fig 3a, 3c, Sup Fig 3, Sup Fig 6, Sup Fig 7, Sup Fig 8:
Only samples with paired body sites were selected.

Fig 4:
Samples were excluded where they have a read count under the subsample read threshold for each paired comparison as described in Methods.

Replication

These results can be replicated using code available https://github.com/blue-moon22/resistomeData using R package https://github.com/blue-moon22/resistomeAnalysis.

Randomization

All figures:
After exclusion of samples, all remaining samples were used or samples were randomly selected from all remaining samples (as detailed below). We had limited metadata for the participants from previous studies, which included age and sex. Sex was not a co-founder in ARG abundance, which we verified using logistic regression modelling. We could not verify whether or not age was a co-founder in ARG abundance between cohorts as age metadata were not available from the US cohort and some Philippines samples. Therefore, we avoided making statistical comparisons between cohorts.

Fig 1 and Sup Fig 1:
18 samples were randomly chosen from each cohort for saliva, dental and stool that had a read account of over the subsample read threshold 6.9, 18 and 16.9 million, respectively.

Blinding

Blinding was not relevant to this study as it relied on available open source datasets from seven previously published studies.

Reporting for specific materials, systems and methods

We require information from authors about some types of materials, experimental systems and methods used in many studies. Here, indicate whether each material, system or method listed is relevant to your study. If you are not sure if a list item applies to your research, read the appropriate section before selecting a response.

Materials & experimental systems

n/a	Involved in the study
☒	☐ Antibodies
☒	☐ Eukaryotic cell lines
☒	☐ Palaeontology
☒	☐ Animals and other organisms
☒	☐ Human research participants
☒	☐ Clinical data

Methods

n/a	Involved in the study
☒	☐ ChIP-seq
☒	☐ Flow cytometry
☒	☐ MRI-based neuroimaging
